# Bullous Hemorrhagic Dermatosis Induced by Enoxaparin: About a Case in Madagascar

**DOI:** 10.1155/2023/5710870

**Published:** 2023-11-02

**Authors:** Fenohasina Rakotonandrasana, Fandresena Arilala Sendrasoa, Andrianandrianina Mbolatiana Kiady Armando Rakotomanana, Herin'Ny Fitiavana Princia Andriatahina, Voahanginirina Nathalie Ralimalala, Samson Léophonte Ramily, Moril Sata, Onivola Raharolahy, Malalaniaina Andrianarison, Irina Mamisoa Ranaivo, Lala Soavina Ramarozatovo, Fahafahantsoa Rapelanoro Rabenja

**Affiliations:** ^1^Department of Dermatology, Faculty of Medicine, University of Antananarivo, Antananarivo 101, Madagascar; ^2^Department of Dermatology, Faculty of Medicine, University of Toamasina, Toamasina 501, Madagascar

## Abstract

Bullous hemorrhagic dermatosis is an adverse reaction occurring within 5 to 21 days after anticoagulation; the diagnosis is to be evoked in the presence of hemorrhagic bullous lesions at a distance from the injection site in the days following the introduction of anticoagulant; this is a diagnosis of exclusion. It is a rare pathology that mainly affects the elderly. A 54-year-old man presented with bullous hemorrhagic lesions on the left upper limb starting at the 4th day after enoxaparin injection, diagnosed as a bullous hemorrhagic dermatosis induced by enoxaparin. We report the first case of bullous hemorrhagic dermatosis induced by enoxaparin in Madagascar.

## 1. Introduction

Bullous hemorrhagic dermatosis (BHD) is an adverse reaction occurring within 5 to 21 days after anticoagulation [1, 2]. It is a rare pathology that mainly affects the elderly, with an incidence of 7.5% [3]. It resolves spontaneously but requires an appropriate local treatment and strict monitoring [4]. We report a case of bullous hemorrhagic dermatosis induced by enoxaparin in a 54-year-old man in Madagascar.

## 2. Case Report

**Figure 1:**
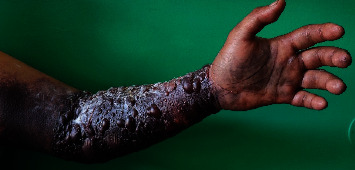
Subcutaneous hematoma associated to hemorrhagic bullae on the front side of the left forearm on day 8 of enoxaparin administration (source: Department of Dermatology, Antananarivo).

**Figure 2:**
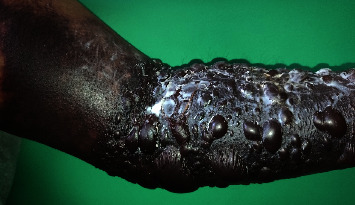
Hemorrhagic bullae on the front side of the left forearm on day 8 of enoxaparin administration (source: Department of Dermatology, Antananarivo).

A 54-year-old man with a history of hypertension and asthma was admitted for painless, non-itchy bullous hemorrhagic lesions evolving in the left upper limb, four days after injection of enoxaparin at prophylactic dose, during hospitalization for infectious pneumonitis. The symptomatology evolved in an apyretic context, without involvement of either the contralateral limb or the mucous membranes. Physical examination revealed large subcutaneous hematoma, extending from the palmar surface to the lower 1/3 of the left arm, associated to several tense bullae with hemorrhagic content, of variable size and posterosive lesions (Figures 1 and 2). No other associated sign was observed.

Laboratory tests showed leukocytosis at 18.09 G/l with a neutrophilia at 13.21 G/l associated with a sedimentation rate at 26 mm/h and a CRP at 11 mg/l, liver and kidney tests were normal, PCR test was negative, blood culture was negative, the coagulation balance sheets revealed a prothrombin at 70%, INR was normal, and the partial thromboplastin time was normal. Skin biopsy was not considered because of the hemorrhagic risk. As diagnostic hypotheses, we thought of gas gangrene and non-necrotizing bacterial dermal and hypodermal dermatosis, but there were no clinical arguments in favor. The elimination of an autoimmune bullous dermatosis was made in the presence of the negativity of the anti-basement membrane and anti-intercellular substance autoantibodies. Doppler ultrasound of the left upper limb ruled out a possible deep vein thrombosis. We retained as diagnosis a bullous hemorrhagic dermatosis induced by enoxaparin after a test of imputability.

As treatment, the patient had local care with antiseptics and tranexamic acid dressing. Combined with a change to rivaroxaban, all heparin-related medications were stopped and notified to the Ministry of Public Health.

**Figure 3:**
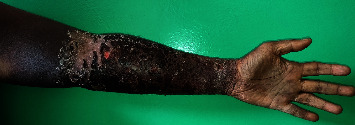
Resorption of hemorrhagic bullous lesions and crusty and scaly lesions of the left forearm on the 12th day (source: Department of Dermatology, Antananarivo).

After 12 days of hospitalization and discontinuation of heparin therapy, the complete resorption of the hemorrhagic bullae and the reduction in skin discoloration were observed in the left upper limb (Figure 3).

## 3. Discussion

Bullous hemorrhagic dermatosis is a rare bullous eruption induced by low-molecular-weight heparin therapy (LMWH); it was first described in 2004 by Dyson and collaborators, in 2 cases of BHD induced by sodic heparin [5]. More than 150 cases have been reported in the literature since the first description. The diagnosis of bullous hemorrhagic dermatosis induced by heparin is a diagnosis of elimination; it should be considered after the elimination of hemostasis disorders and autoimmune bullous dermatosis [6].

Our case is consistent with the literature; bullous hemorrhagic dermatosis is characterized by bullous lesions with hemorrhagic content, which are painless, non-itchy, and frequently located at a distant location from the injection site, particularly on the limbs [7]. A study of 91 cases of BHD in 2018 by Russo and collaborators showed the absence of pain and pruritus. They also reported a development of cutaneous manifestations from a few hours to 9 months after the start of the anticoagulant [4].

Concerning the topography, Perrinaud and collaborators had described through 3 cases of BHD a remote attack of the injection site [8]. 5 cases were reported by Maldonado and collaborators, in which they noted a lesion distant from the injection site and the most concerned topographies were the limbs, followed by the trunk [9].

On the therapeutic level, many authors have reported in their studies a favorable evolution after discontinuation of heparin therapy [10]. The use of rivaroxaban was opted for the relay of his treatment with heparin, and a favorable evolution was observed in our patient after 12 days of stopping heparin therapy. The literature has reported that maintaining anticoagulation with LMWH would increase the risk of recurrence [4, 11, 12] and that the resolution of hemorrhagic bullae depends on the dose of LMWH [4, 11].

## 4. Conclusion

Hemorrhagic bullous dermatosis under heparin is a rare, non-dose-dependent adverse effect. It is a benign affection, but with a risk of disturbance of the hemodynamic parameters. The physiopathological mechanism is still unknown, but it is without immunological mediation and disappears on its own after the discontinuation of the responsible anticoagulant. Few studies have been conducted in Africa; this is the first case report of bullous hemorrhagic dermatosis from Madagascar.

## Abbreviations

CRP: C-reactive protein.
